# Structural basis of bidirectional allostery across the heme in a cytochrome P450 enzyme

**DOI:** 10.1016/j.jbc.2023.104977

**Published:** 2023-06-29

**Authors:** Amit Kumar, D. Fernando Estrada

**Affiliations:** Department of Biochemistry, Jacobs School of Medicine and Biomedical Science, University at Buffalo, Buffalo, New York, USA

**Keywords:** cytochrome P450, nuclear magnetic resonance (NMR), allosteric regulation, protein cross-linking, spectroscopy, conformational change, ligand binding

## Abstract

Cytochromes P450 (CYPs) are heme-containing enzymes that are present in all kingdoms of life and share a structurally homologous, globular protein fold. CYPs utilize structures distal to the heme to recognize and coordinate substrates, while the necessary interactions with redox partner proteins are mediated at the opposite, proximal surface. In the current study, we investigated the functional allostery across the heme for the bacterial enzyme CYP121A1, which utilizes a non-polar distal-to-distal dimer interface for specific binding of its dicyclotyrosine substrate. Fluorine-detected Nuclear Magnetic Resonance (^19^F-NMR) spectroscopy was combined with site-specific labeling of a distal surface residue (S171C of the FG-loop), one residue of the B-helix (N84C), and two proximal surface residues (T103C and T333C) with a thiol-reactive fluorine label. Adrenodoxin was used as a substitute redox protein and was found to promote a closed arrangement of the FG-loop, similar to the addition of substrate alone. Disruption of the protein–protein interface by mutagenesis of two CYP121 basic surface residues removed the allosteric effect. Moreover, ^19^F-NMR spectra of the proximal surface indicate that ligand-induced allostery modulates the environment at the C-helix but not the meander region of the enzyme. In light of the high degree of structural homology in this family of enzymes, we interpret the findings from this work to represent a conserved allosteric network in CYPs.

Cytochromes P450 (CYPs) comprise a superfamily of structurally homologous heme-containing enzymes that play critical roles in drug and xenobiotic metabolism, along with the biosynthesis of endogenous steroids and other natural products (1, 2, 3, 4, 5). CYP catalysis requires the formation of a high-valent iron-oxo intermediate (compound I - FeO^3+^), which is driven by the transfer of two external electrons. Class-I CYPs, which comprise all microsomal membrane-associated enzymes and include most drug-metabolizing systems, are reduced following an interaction with an NADPH-dependent P450 oxidoreductase (6). In contrast, Class-II CYPs include all mitochondrial and most bacterial enzymes and are reduced by soluble redox partner ferredoxins like Adrenodoxin (Adx) and Putidaredoxin (Pdx) (7, 8, 9).

The protein–protein interactions between the proximal surface of CYPs and either redox partners or modulator proteins are a known contributor to CYP function beyond the mere delivery of electrons (10, 11, 12). For example, the efficiency of the lyase reaction performed by the bifunctional steroidogenic enzyme CYP17A1 is specifically enhanced (over its hydroxylase activity) by the addition of the modulator protein cytochrome *b*_*5*_ (13, 14). Competitive binding between *b*_*5*_ and CYP17A1 with P450 oxidoreductase was also found to be influenced by the presence of the particular CYP17A1 substrate (15, 16). Similarly, cytochrome *b*_*5*_ also stimulates the activity of the xenobiotic enzymes CYP2E1, CYP3A4, and CY2A6 (17, 18, 19, 20). In Class-II CYPs, recent studies of the mitochondrial enzyme CYP24A1 indicate that the redox partner protein Adx, when combined with the CYP24A1 vitamin-D substrate, exerts a coordinated effect on the enzyme structure that is directed at both the distal ligand-binding surface and the C-D helical turn of the proximal surface (21). Such allostery across the heme is also evident in studies of the effect of Adx on the ligand behaviors of the glucocorticoid metabolizing enzyme CYP11B1 (22). Additional examples are provided by analysis of Pdx-induced structural modulation of CYP101 (P450cam) of *Pseudomonas putida* (9, 23). Moreover, redox partner-induced modulation of CYP structure appears to occur in a bidirectional manner since substrate addition also impacts molecular recognition of the redox partner. The addition of substrate, for example, has been demonstrated to affect the affinity or contact surfaces of particular CYP-redox proteins (11, 17, 24).

While such structural cross-talk has become an established feature of CYPs, the particular surfaces involved are poorly understood. Analyses of allostery in CYPs have thus far been primarily focused on understanding the basis of homotropic cooperativity resulting from multiple substrate coordination sites (25, 26, 27, 28, 29). The enclosed study investigates allostery across the heme for CYP121A1 (CYP121), an essential enzyme from *Mycobacterium tuberculosis* (30). CYP121 mediates the formation of an intramolecular carbon-carbon bond between the phenol groups of dicyclotyrosine (cYY) to form mycocylosin, a compound of as yet undermined function (31, 32, 33). A unique feature of CYP121 is its reliance on a distal-to-distal dimer interface, in which the substrate-sensitive FG-loop regions of the respective monomers are positioned on opposite sides of the dimer and form part of the non-polar interface. Disruption of this dimer interface results in a loss of specificity for cYY, and therefore, a significant decrease in catalysis occurs (34). Interestingly, the unique arrangement of CYP121 dimers resembles the membrane insertion topology of the catalytic CYP domain for mammalian enzymes (35). In both cases, the non-polar distal surface, and the FG-loop in particular, is constrained by either the presence of a membrane bilayer or, in the case of CYP121, the presence of another hydrophobic distal surface. In this way, CYP121 represents an informative model system for understanding allostery in structurally homologous mammalian enzymes in which molecular motions at the distal surface are constrained, but with the advantage of robust protein expression, superior protein stability, and the absence of detergent molecules that would complicate a similar analysis in membrane-extracted enzymes.

Here, we utilize ^19^F-NMR and site-selective incorporation of thiol-reactive fluorine labels to monitor long-range structural changes that occur in response to either cYY or bovine Adx. While the cognate Mtb redox partner for CYP121 remains unknown, Adx supports catalysis of the enzyme and therefore forms a functionally relevant CYP-ferredoxin complex. The S171C mutation was used to monitor conformational selection at the FG-loop as has been reported previously (34, 36). The additional mutations T103C and T333C were incorporated separately to monitor distinct surfaces on the proximal surface, representing the C-helix and the CYP meander region, respectively. Two key findings from this work are (i) that cYY and non-specific azoles induce a structural change near the C-helix, but not the meander region of the CYP121 proximal surface and (ii) that redox partner binding promotes a closed state of the distal-facing FG-loop. This latter finding was confirmed using a negative control consisting of a double arginine mutation of CYP121 (R95E_R352E) that does not bind Adx. These findings are discussed in a broader context and in terms of how they advance our understanding of long-range CYP allostery.

## Results

### Redox partner induces a substrate-bound conformation of CYP121

**Figure 1:**
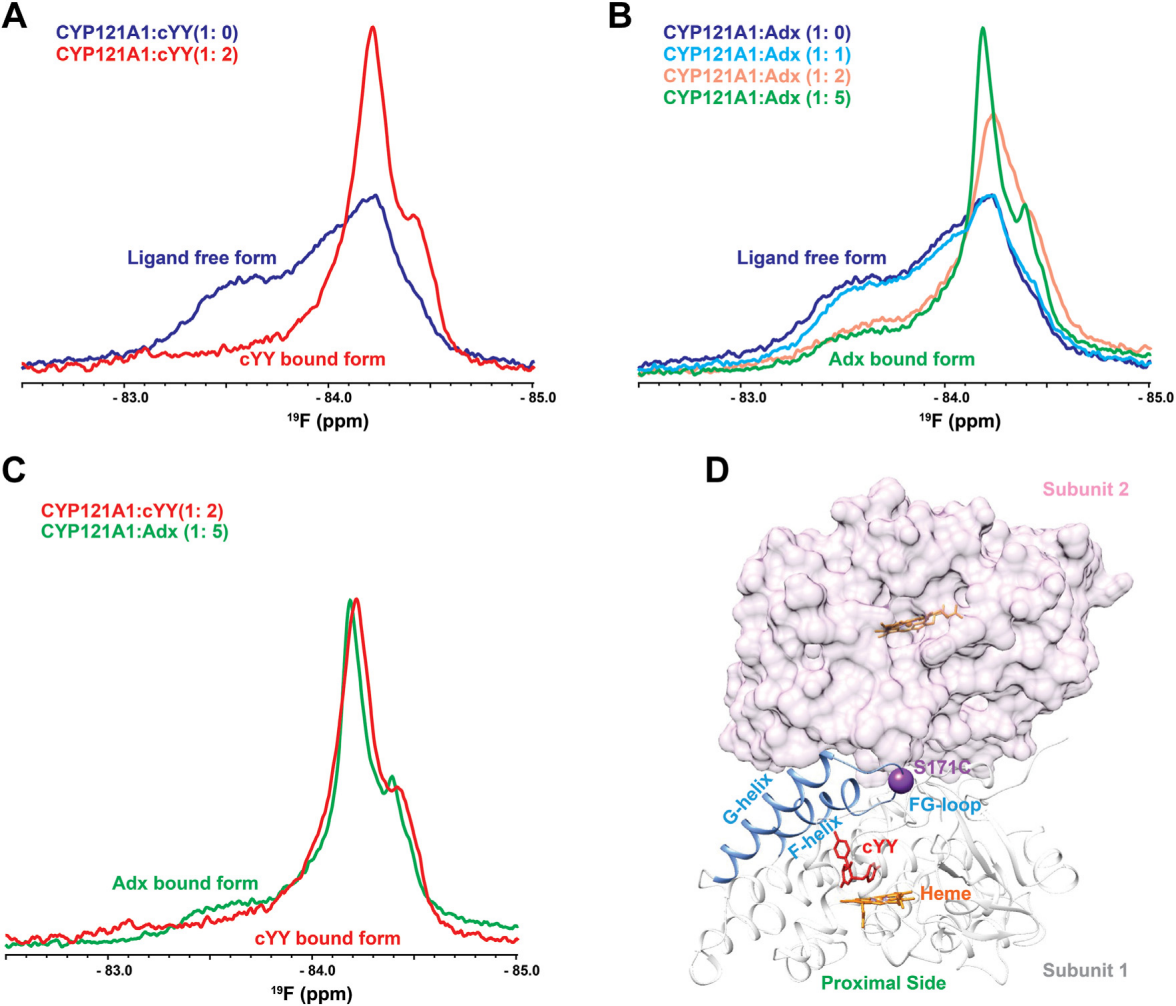
**Substrate and Adx-induced modulation of the**^**19**^**F labeled CYP121 FG-loop.** Decreased heterogeneity of the multiple ligand-free conformers at the FG-loop is induced by cYY (*A*), as well as by titration of the redox protein Adx (*B*). The Adx-bound and cYY-bound spectra are overlaid in panel (*C*). The spatial relationship between the proximal redox protein-binding surface and the S171C label on the FG-loop is shown in panel (*D*), with the second molecule from the CYP121 functional dimer depicted as a surface representation.

The intervening loop between the F and G α-helices (the FG-loop) borders the distal end of all CYP active sites and undergoes significant remodeling in response to ligands (34, 36, 37). Repositioning this loop may result in forming or closing specific ligand access channels. Previous reporting on CYP121 suggests that the FG-loop of the enzyme is heterogenous in the absence of ligand but forms a single ligand-bound conformation when in complex with either cYY or azole-containing CYP inhibitors (34, 36, 37). This effect was reproduced for the current study and is demonstrated in the overlaid ^19^F-NMR spectra in Figure 1*A*. Here, the FG-loop has been labeled by incorporation of a cysteine at residue 171, followed by incubation with the thiol-reactive label BTFA. The broad downfield resonance near −83.6 ppm is no longer present in the cYY-bound spectrum, while the larger peak near −84.4 ppm increases in intensity and narrows in linewidth. In the previous studies the addition of other ligands was reported to induce similar, but not identical, changes in the spectrum, thus indicating that the downfield resonance represents a ligand-free arrangement of the FG-loop.

In the present study, the ^19^F labeled FG-loop was monitored in the absence and presence of the mammalian ferredoxin Adx. While the cognate redox partner from Mtb remains undetermined, Adx supports the catalysis of CYP121, and its chemical cross-linking (reported later in this study) produces a single band consistent with a 1:1 CYP-Adx complex. Interestingly, the addition of Adx was observed to reduce the intensity of the downfield, ligand-free resonance of the loop, while increasing the intensity of the upfield resonance, despite the absence of cYY in the experiment (Fig. 1, *B* and *C*). The similarities in the spectra of cYY-bound and Adx-bound protein include a minor upfield peak that forms near −84.3 ppm and are overlaid together in Figure 1*C*. These similarities indicate that the addition of substrate to CYP121 and formation of the complex with the redox partner separately induce similar arrangements of the FG-loop. Moreover, the data are also consistent with the presence of long-range (proximal-to-distal) communication in the CYP121 structure, as the redox binding surface (Fig. 1*D*) is located approximately 30 Å from the labeled residue S171C.

### Site selection of structural probes of the CYP121 proximal surface

**Figure 2:**
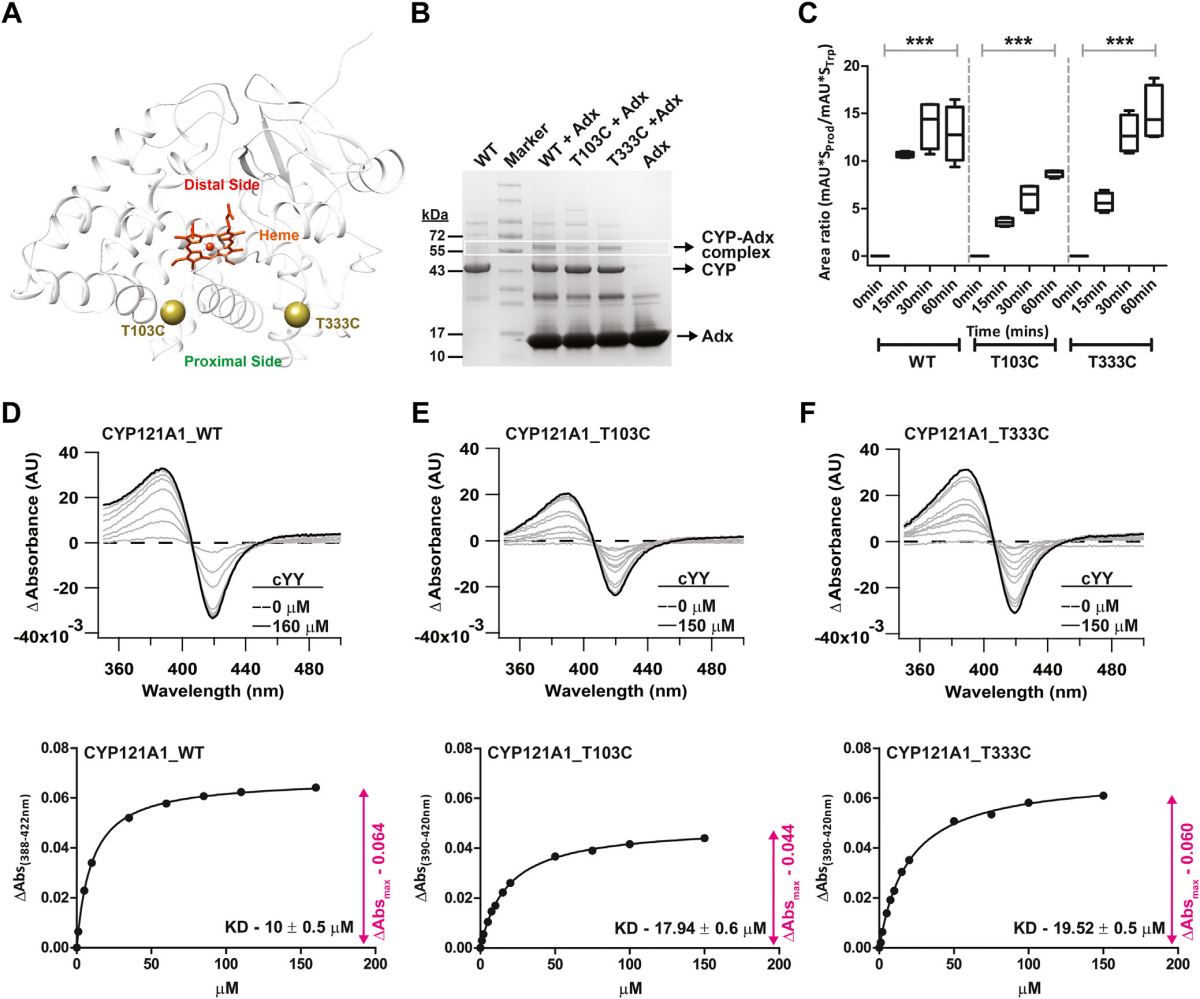
**Functional characterization of T103C and T333C mutations of CYP121.** The two sites selected for ^19^F-NMR labeling are nearly equidistant from the heme iron center (*A*). Both mutants are capable of binding to Adx and supporting electron transfer, as indicated by EDC cross-linking (*B*) and reconstituted functional assays (*p* < 0.05) (*C*). Panels (*D*–*F*) summarize type-I absorbance difference spectra and saturation plots resulting from titrations with cYY. The T103C mutant results in a modest decrease in both total cYY converted to mycocyclosin as well as the maximum change in absorbance. All binding data were acquired in triplicate and fit using the one-site binding equation. Error bars are smaller than data point markers.

The long-range conformational changes detected in the FG-loop upon the addition of redox protein are supportive of functional allostery. Specifically, the addition of Adx removes the ligand-free conformation of the loop. To investigate whether such modulation of CYP structure occurs in a bidirectional manner, it was necessary to identify structural probes on the proximal surface of the protein that may then be monitored by ligand binding at the distal site. The two sites selected for ^19^F-labeling, Thr-103 and Thr-333, were selected based on (i) avoidance of positively charged residues, since CYP, redox protein interactions rely on the complementarity of charges for binding, (ii) a similar polar side chain chemistry as that of cysteine, and (iii) representation of structural features of the proximal surface that are common to all CYP structures. The two residues are on opposite sides of the central heme coordination site (Fig. 2*A*), with Thr-103 located near the C-helix and Thr-333 located on the meander region. Cysteine mutations in these locations were incorporated separately from each other and separately from the S171C mutation to simplify the interpretation of the spectra. Mass spectrometry quantification from prior labeling experiments indicated that the native cysteine residues Cys-147 and Cys-51, which are not surface residues, are not efficiently labeled by BTFA (36). The third native cysteine (Cys-345) is presumed to not be available for labeling due to its coordination of the heme.

It was first necessary to confirm that the T103C and T333C mutations were functionally viable. The ability of the mutants to form a complex with Adx was confirmed by cross-linking using EDC. The cross-linked product for each protein and mutant complex with Adx is shown in Figure 2*B*. Next, the ability of the mutants to catalyze the cYY phenol coupling reaction in a time-dependent manner was measured using a reconstituted electron delivery system composed of bovine Adx and AdR. The plots in Figure 2*C* report the accumulation of mycocyclosin over 15-, 30-, and 60-min increments. Both mutants showed slower accumulation of the product in comparison to the wild-type CYP121. However, while T103C reduced overall product formation by approximately 25%, T333C activity at 60 min was comparable to that of the native enzyme. Last, substrate binding for the mutants was compared with wild-type CYP121 using absorbance difference assays, which report on CYP-substrate interactions by quantification of changes in the spin state of the heme (Fig. 2, *D*–*F*). The calculated Kd values of 18 μM and 20 μM for T103C and T333C are similar to the wild-type Kd of approximately 10 μM. It was noted, however, that the T103C mutation modestly decreases the net change in absorbance (Δ Abs_max_).

### Assessing the effect of paramagnetism on ^19^F-T103C and ^19^F-T333C

**Figure 3:**
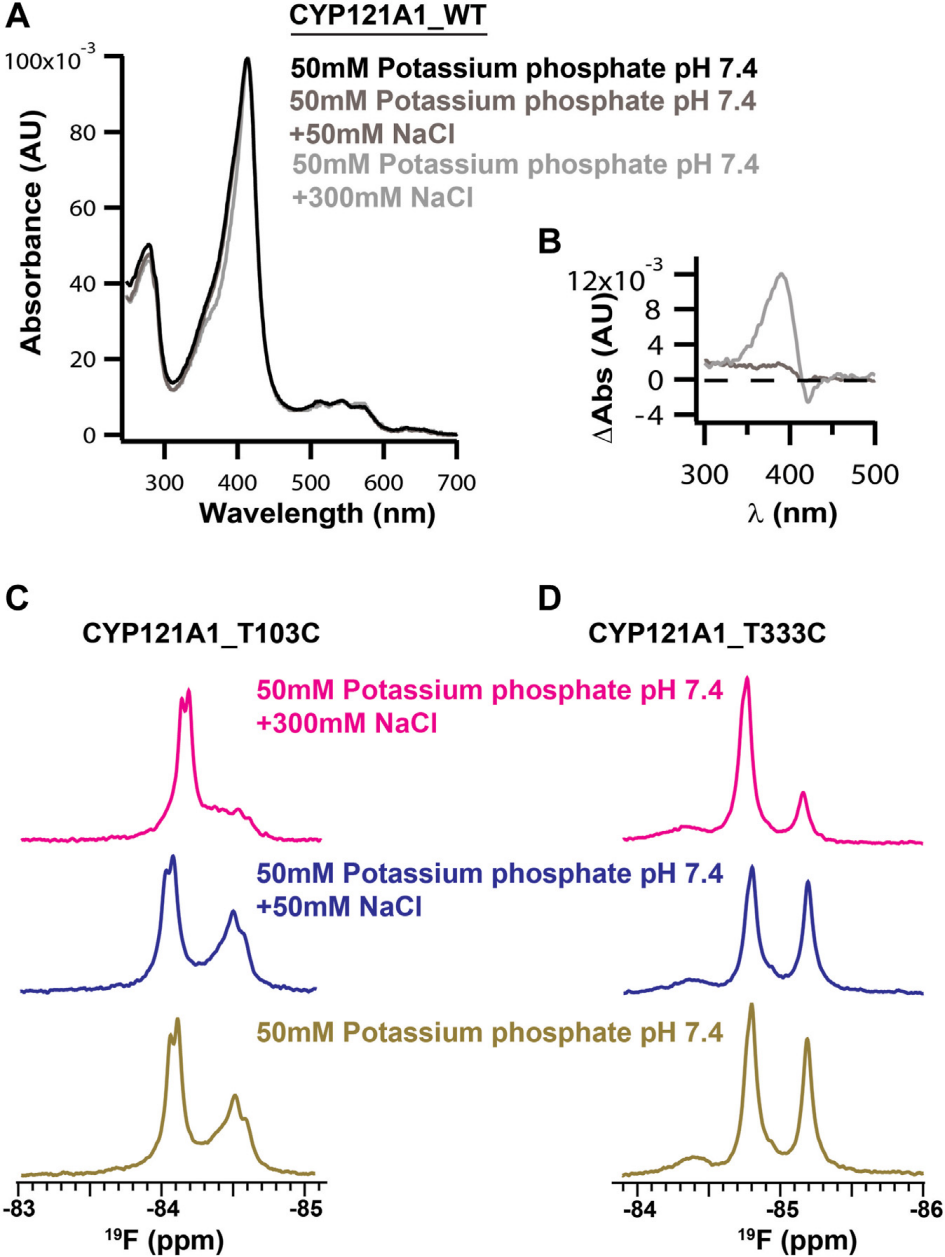
**The NMR spectra of**^**19**^**F-T103C and**^**19**^**F-T333C are sensitive to the heme spin state.** The spin state of the CYP121 heme is modulated by the ionic strength of the buffer solution (*A*), and the high-salt to low-salt difference spectrum is shown in (*B*). Salt modulation of the spin state was then used to confirm the presence of paramagnetically induced pseudo-contact shifts in ^19^F-NMR spectra of each proximal surface probe. At 50 mM potassium phosphate, both spectra contain two primary resonances (panels *C* and *D*). For each label, acquisitions in the presence of increasing NaCl (which promotes low spin) decrease the upfield resonance.

1D ^19^F spectra representing BTFA labeled T103C and T333C were acquired first in the absence of ligand in order to ascertain the effect of paramagnetic relaxation or pseudo-contact shifts that may result from the neighboring heme. Unlike the NMR signal of the distal FG-loop, which is located farther from the heme and is therefore independent of changes in the heme spin state, the ^19^F nuclei on the proximal surface are located a mere 12 and 14 Å from the iron center. Here, the salt sensitivity of the enzyme’s spin state was used to modulate between low and high spin forms of the heme and therefore served as a marker for the effects of changing spin states on the proximal surface labels. Ligand-free wavelength absorbance spectra of CYP121 (Fig. 3*A*) indicate that under low salt conditions (50 mM potassium phosphate pH 7.4), CYP121 retains an intrinsic amount of high-spin character, represented by broadening of the Soret band near 390 nm. The active site then converts entirely to low-spin at higher salt concentrations (50 mM potassium phosphate pH 7.4 + 300 mM NaCl). Therefore, NMR spectra of each labeled mutant were compared using independent samples prepared at increasing salt concentrations. The baseline spectrum of ^19^F-T103C without NaCl contains two primary resonances at −84 ppm and −84.5 ppm (Fig. 3*C*). Increasing NaCl (therefore increasing low-spin) induces the narrow downfield resonance to move upfield by 0.10 ppm, while the broader upfield resonance gradually decreases in intensity. This response indicates that at least some of the heterogeneity evident in the 1D spectrum results from pseudocontact shifts rather than structural heterogeneity. A similar effect is observed for ^19^F-T333C (Fig. 3*D*), where an upfield resonance decreases in intensity at higher NaCl concentrations but, in this case, is paired with increased intensity in the downfield resonance. While salt-induced structural changes cannot be ruled out as a cause for the changes in the NMR spectra, these results nonetheless indicate that the proximity of the label to the paramagnetic center and the possibility for pseudocontact shifts will need to be taken into consideration when evaluating ligand-bound spectra.

### Substrate modulates the proximal surface of CYP121

**Figure 4:**
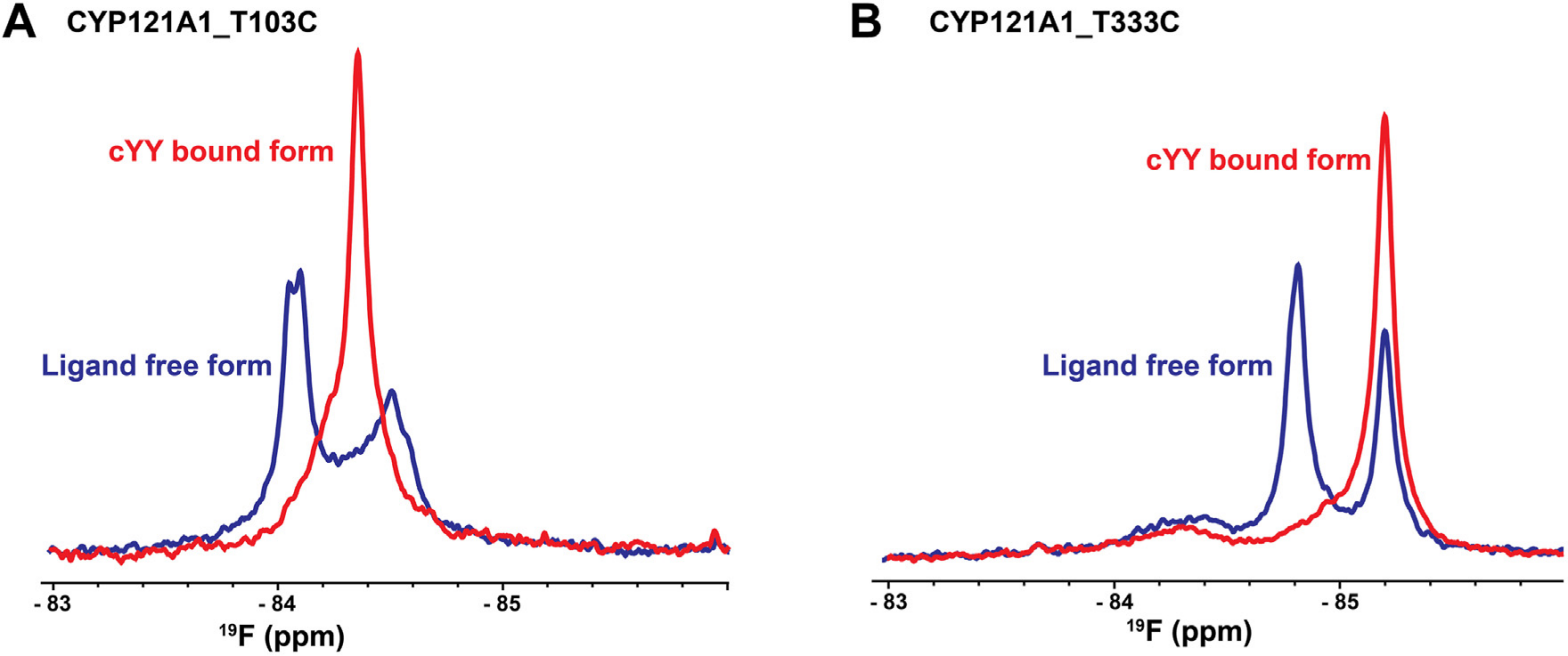
**cYY-induced modulation of the CYP121 proximal surface.**^19^F-NMR Spectra acquired in the presence of two-fold excess cYY result in the introduction of a new peak at −84.3 ppm for CYP121_T103C (*A*). However, in the case of CYP121_T333C, only changes in the paramagnetic pseudocontact shifts are observed (*B*).

Having established the baseline for ^19^F-NMR spectra of the proximal surface of CYP121, data sets of ^19^F-T103C and ^19^F-T333C were acquired at an intermediate salt concentration (50 mM potassium phosphate and 50 mM NaCl) and in the presence of a twofold addition of cYY. An overlay of the spectrum of cYY-bound and ligand-free ^19^F-T103C shows a new signal centered at −84.3 ppm and a strong reduction in the ligand-free resonances. Notably, the new peak does not align with the upfield signal that correlates with the high-spin form of the protein, even though the addition of cYY in excess does introduce high-spin populations as measured by a change in absorbance. Therefore, the changes in the cYY-bound spectrum of ^19^F-T103C are interpreted to represent a combination of both paramagnetic pseudocontact shifts resulting from the introduction of the high-spin form of the heme as well as the detection of substrate-induced modulation of the protein structure (Fig. 4*A*). In contrast, cYY does not induce the exact change in the spectrum of ^19^F-T333C (Fig. 4*B*). Here, the increase in the upfield resonance and the decrease in the downfield resonance are consistent with salt modulation of the heme spin state as described earlier (Fig. 3, *C* and *D*), thus indicating that changes in the NMR spectrum at this site are explained entirely by changes in the spin state, rather than changes in protein structure. Taken together, these spectra point toward cYY-induced modulation of the proximal surface that is directed toward T103C of the C-helix, while T333C of the meander region is unaffected.

### Azole ligand effects on the proximal surface of CYP121

**Figure 5:**
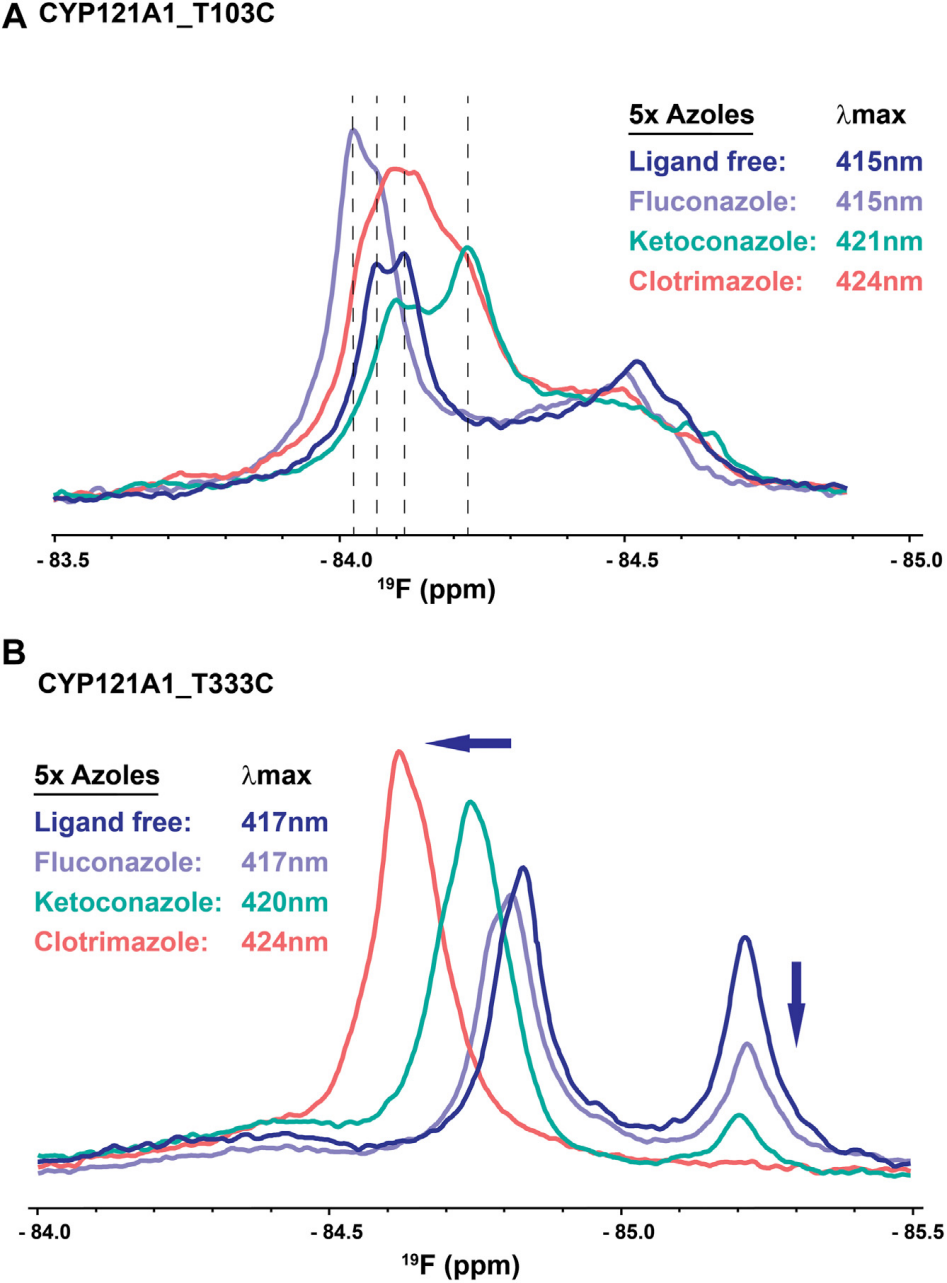
**Azole compound effects on**^**19**^**F-T103C and**^**19**^**F-T333C.** Spectra acquired in the presence of 5-fold excess concentrations of fluconazole, ketoconazole, and clotrimazole were compared to the ligand-free spectrum. Changes in ^19^F-T103C (*A*) are not predicted by the final absorbance values, while changes in ^19^F-T333C (*B*) appear to respond entirely to the spin state of the heme. Absorbance values were measured directly from samples prior to NMR data acquisition.

The ^19^F-T103C and ^19^F-T333C spectra were then used to evaluate ligand-induced modulation of the proximal surface that stems from non-specific ligands rather than cYY. In addition to salt modulation of the CYP121 spin state, the spin state can also be influenced by the introduction of type-II CYP ligands like azoles that are capable of forming a coordinate covalent bond with iron, thus stabilizing a low-spin form of the heme (38). Here, azole-containing compounds were selected that introduce a range of absorption values for the Soret peak, from fluconazole which does not significantly modify absorbance, but is nonetheless known to bind the active site (PDB ID – 2IJ7 3G5H) (39), to ketoconazole which establishes a change in absorbance at 421 nm, to the more significant effect from clotrimazole at 424 nm (Fig. S1). ^19^F-T103C NMR samples that were prepared in the presence of fivefold excess concentrations of each compound produced a series of changes in the final spectra, particularly for the downfield resonance (Fig. 5*A*) that, due to the salt modulation of the NMR spectra, is interpreted to represent a low-spin form of the enzyme. In these experiments, clotrimazole and ketoconazole induced upfield perturbations of the low-spin resonance at −84 ppm, accompanied by broadening of the signal and introducing minor secondary peaks. For example, ketoconazole induced a second distinct peak near −84.3 ppm that aligns with a shoulder of the broader clotrimazole-bound peak. Fluconazole, which is known to bind CYP121 *via* multiple modes, induced an opposite downfield perturbation from the ligand-free resonance but retained a shoulder that aligns closely with the clotrimazole-bound peak. Importantly, the extent of the spectral perturbations in the low-spin peak is not predicted by the final absorbance value of the Soret peak. In contrast, the ^19^F-T333C spectra (Fig. 5*B*) do reflect a step-wise downfield shift in the corresponding NMR signal and a decrease in the smaller upfield peak at −84.5 ppm. Notably, these changes are predicted by the corresponding change in absorbance of the heme, with clotrimazole (absorbance at 424 nm) producing the largest downfield resonance, with nothing remaining of the upfield resonance that was previously assigned as the high-spin form. However, the NMR spectrum of the fluconazole-bound sample (absorbance at 417 nm) closely resembles the ligand-free spectrum. This comparison provides further evidence that the structure near ^19^F-T103C is responsive to changes in ligand binding and orientation, while the transition toward a low-spin heme entirely predicts the perturbations at ^19^F-T333C. The data also indicate that ligand modulation of the proximal surface is not limited to cYY, since differences in ligand size and chemistry also contribute to notable changes at this surface.

**Figure 6:**
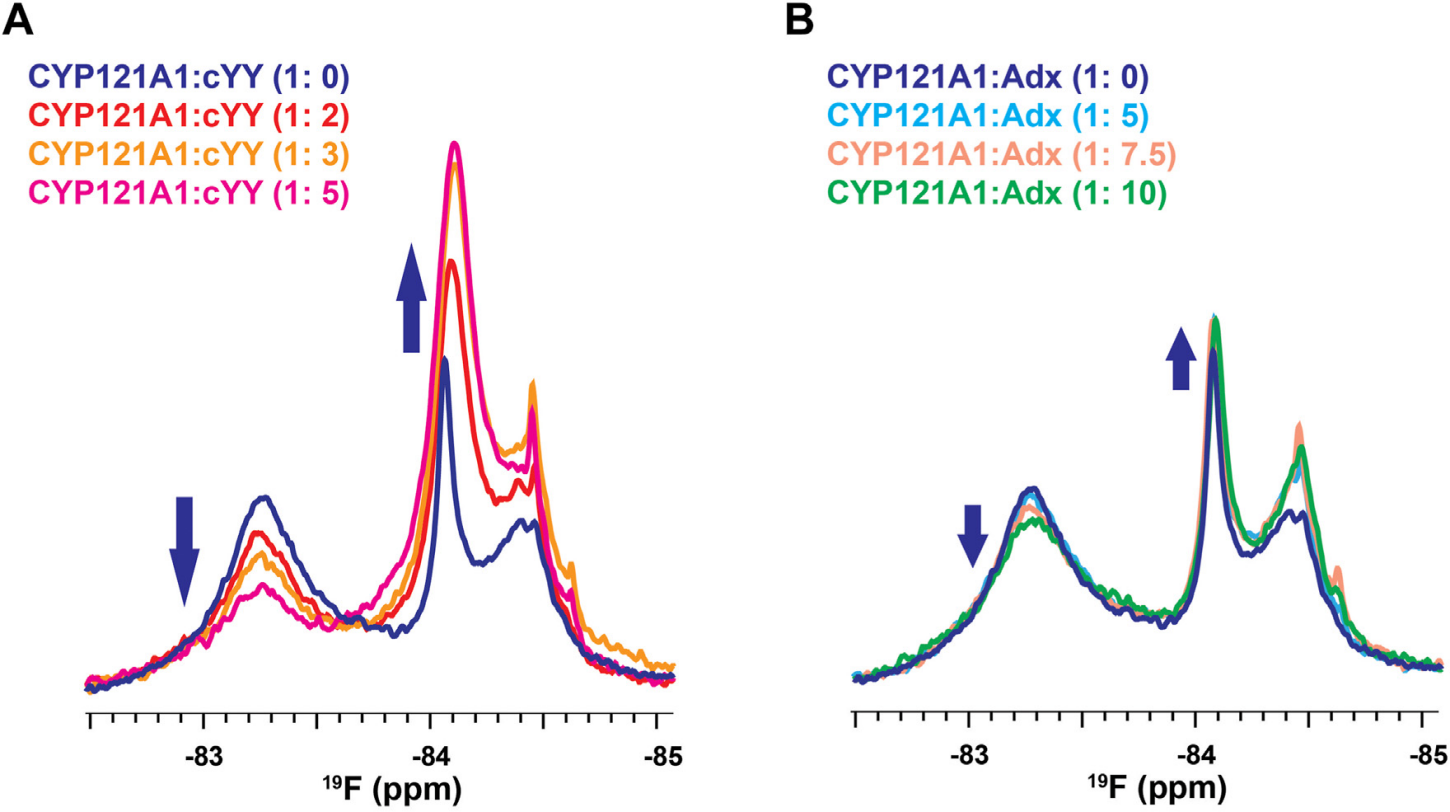
^**19**^**F-NMR spectra of**^**19**^**F-N84C.***A*, titration of ^19^F-N84C with cYY. *B*, titration with excess Adx. The *block arrows* indicate the changing peak intensities in response to either cYY or Adx.

The discovery of proximal to distal allostery that occurs near residue T103, but not T333 (Figs. 4 and 5), implies that the likely route of information includes the B-helix of CYP121. The B-helix and the associated BC-loop are already theorized to participate in substrate coordination due to the presence of Asn-85, which is known to hydrogen bond with the diketopiperazine ring of cYY (30). To investigate the substrate and redox partner effects along a likely intermediate region of the allosteric pathway, the solvent-oriented residue Asn-84 was mutated to cysteine and labeled with BTFA in a similar fashion as the proximal residues T103C and T333C. However, a functional evaluation of the N84C mutant indicates that the enzyme has lost a significant amount of catalytic activity (Fig. S3). Notably, absorbance difference measurements with cYY indicate a threefold loss of substrate affinity, with a minimal high-spin form of the heme as a result. Despite compromised cYY binding, NMR spectra were recorded in the presence of cYY, Adx, or various azole-containing inhibitors. Interestingly, the perturbations induced by cYY are consistent with those observed at the FG-loop. The initial ^19^F-N84C spectrum reflects conformational heterogeneity that is reduced upon titration with cYY (Fig. 6*A*); a downfield resonance loses intensity in favor of increased intensity in the upfield peak (analogous to the distribution of states in the FG-loop region). The weaker absorbance response from cYY in this mutant is consistent with the weaker binding apparent in the NMR data. Additionally, while the spectral changes in ^19^F-N84C resulting from titration with Adx are modest (Fig. 6*B*), the Adx-induced perturbations occur in a similar fashion as those induced by cYY. Therefore, solution monitoring of the intermediate residue Asn-84 is consistent with involvement of the B-helix in long-range allostery. Such involvement likely also explains the functional sensitivity of mutating a residue at this site.

### Redox partner binding alters the apparent affinity of CYP121 for cYY

**Figure 7:**
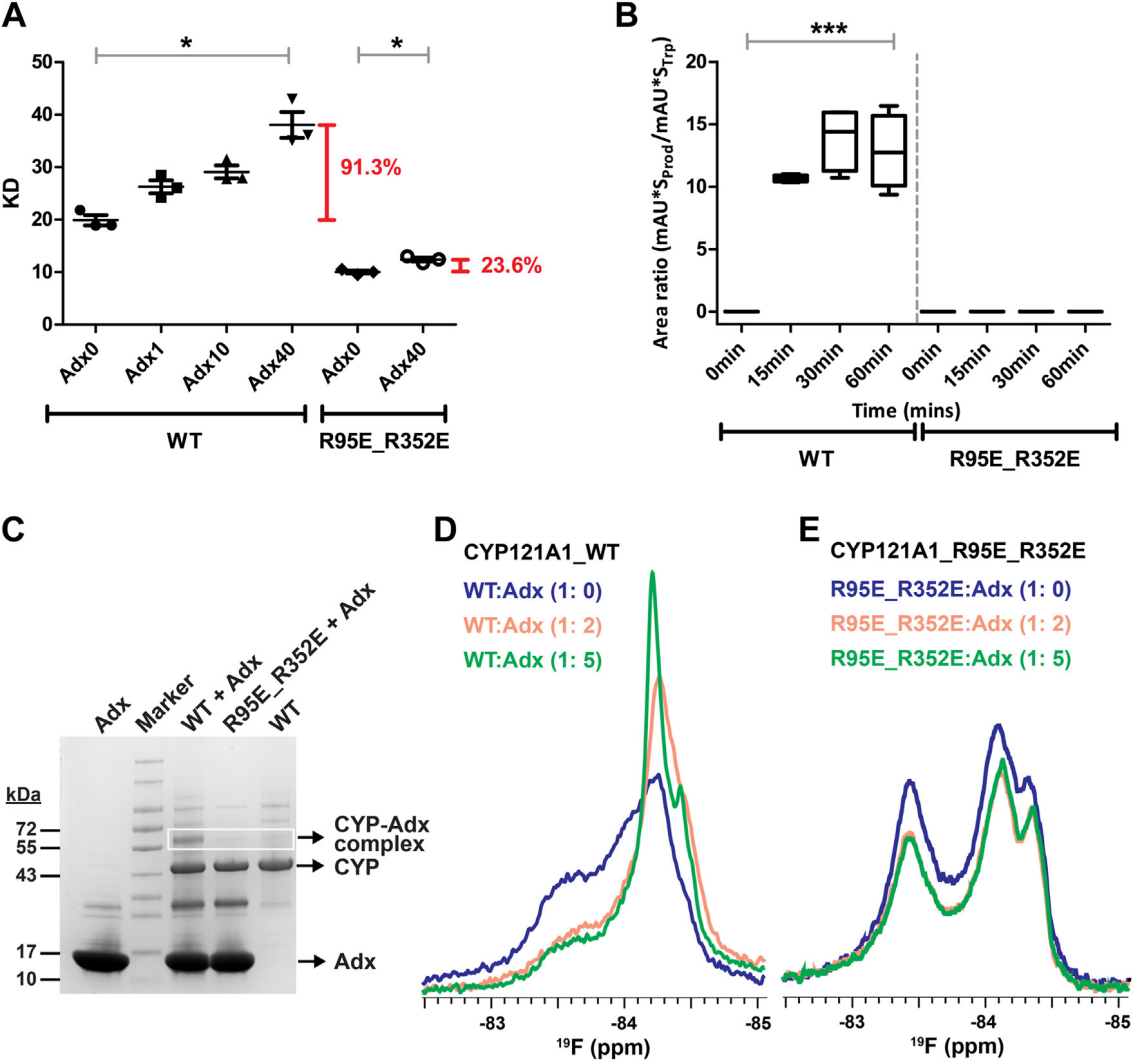
**Proximal-to-distal allostery confirmed by the mutant R95E_R352E.** Titration of Adx interferes with cYY binding by spectral binding assay (*A*), likely by promoting a closed conformation of the enzyme. The significance of Adx0 and Adx40 for both datasets was extracted by *t* test (*p* < 0.05). The double proximal mutant R95E_R352E was designed to prevent the CYP-redox partner complex and did not show catalytic activity (*B*) or the ability to cross-link with Adx (*C*). The double CYP121 mutant was also combined with S171C to ^19^F label and monitor effects at the FG-loop (*D*) and (*E*).

^19^F-S171C spectra acquired in the presence of Adx indicate that the addition of redox partner decreases the presence of a ligand-free conformation of the distal FG-loop (Fig. 1, *B* and *C*). This would be consistent with a closing of the active site that can be induced either by cYY or by the redox partner alone. Such a model would predict that the pre-formation of the CYP121-redox protein complex would bias open and closed equilibria toward a close state, and therefore interfere with cYY binding overall. To address this, absorbance measurements of cYY binding were quantified for wild-type CYP121 in the presence of increasing concentrations of Adx. The resulting Kd values are plotted in Figure 7*A*. The addition of Adx gradually weakens the affinity for cYY, with a 40-fold excess of Adx resulting in a near doubling of the apparent Kd to approximately 40 μM.

Next, to address the possibility that this change in affinity may instead result from non-specific crowding of CYP121 in the presence of large relative concentrations of Adx, a double arginine mutation was designed as a negative control for the formation of the protein complex. The CYP121 mutant R95E_R352E was designed based on a multiple sequence alignment of all Mtb CYPs and a selection of the most highly conserved basic charges on the proximal surface. The basic charges were reversed to negatively charged glutamate residues to ensure disruption of the complex. The double mutant does not support cYY catalysis (Fig. 7*B*), and EDC cross-linking does not show a cross-linked product between CYP121 and the mutant R95E_R352E (Fig. 7*C*). While R95E_R352E on its own does display a higher affinity for cYY, a 40-fold addition of Adx produces only a modest increase in the apparent Kd; a noticeably smaller effect than was observed for Adx addition to wild-type CYP121. This comparison suggests that redox partner protein promotes a closing off of the CYP121 active site, as predicted by the ^19^F-S171C spectra.

Last, the R95E_R352E double mutant was combined with S171C in order to monitor the ^19^F-NMR spectra of the FG-loop for the version of CYP121 that is incapable of binding a redox partner. It was first observed that the 1D ^19^F-S171C_ R95E_R352E spectrum is distinct from that of the protein with the unmodified proximal surface in that the broad downfield resonance is more resolved than in ^19^F-S171C (Fig. 7, *D* and *E*). However, the addition of 5-fold excess Adx fails to induce perturbations of the FG-loop spectrum (Fig. 7*E*). This negative control essentially confirms that the addition of Adx alters the arrangement of the FG-loop, thus also confirming the presence of bi-directional allostery in the CYP.

### cYY modifies the specificity of the interaction with the redox partner

**Figure 8:**
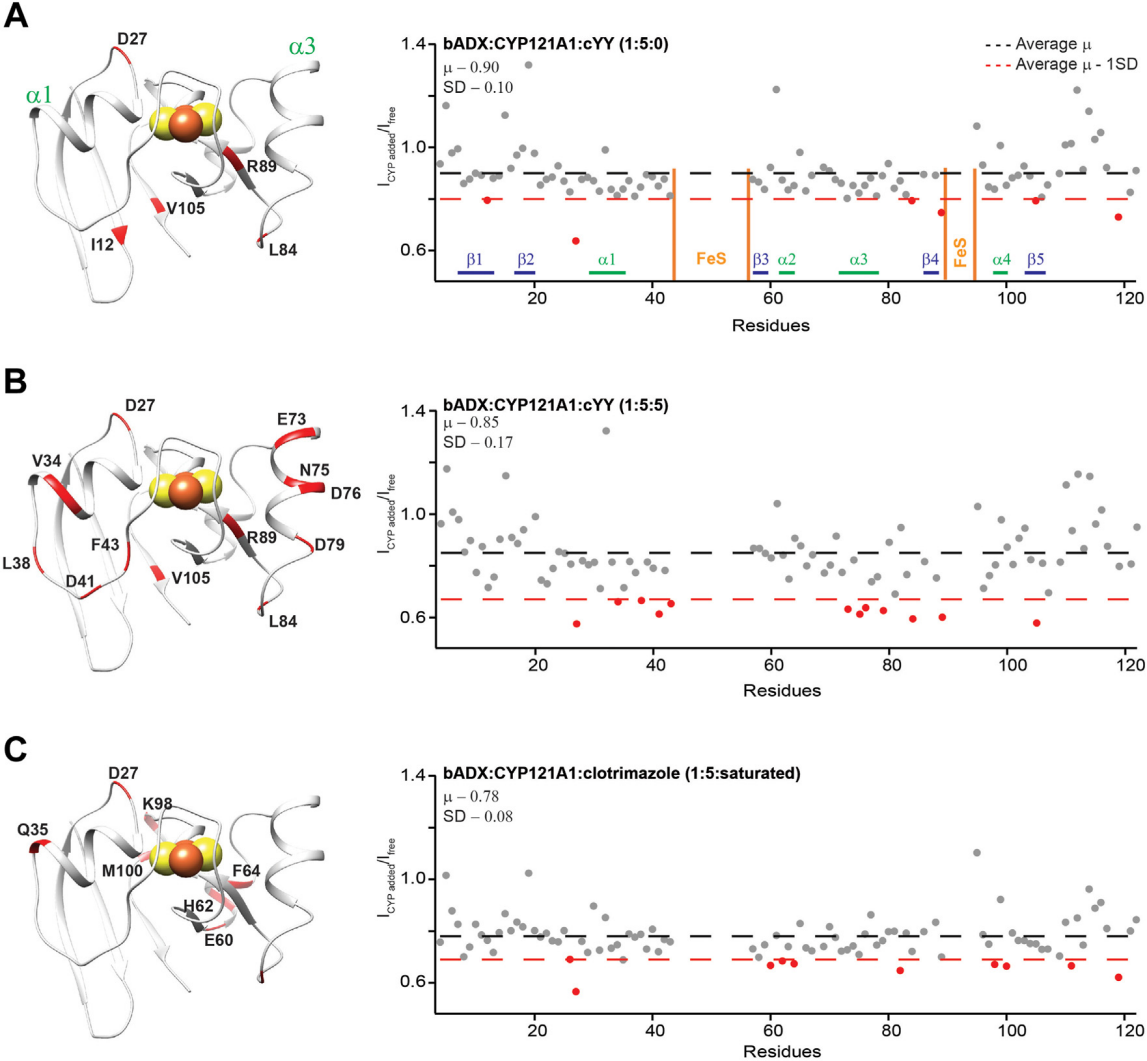
**Substrate binding to CYP121 induces increased specificity in the CYP-Adx complex.** 2D ^1^H-^15^N HSQC spectra of uniformly labeled ^15^N-Adx were acquired alone and in the presence of ligand-bound and ligand-free CYP121. The remaining peak intensity ratios resulting from 5-fold excess additions of ligand-free CYP121 (*A*), cYY-bound CYP121 (*B*), and clotrimazole-bound CYP121 (*C*) indicate a complex with increased specificity that results from the addition of cYY.

cYY binding modulates the structure of the proximal surface of CYP121 near residue Thr-103 (Fig. 4). To determine whether such allostery results in modified binding to the redox protein, the interaction was then investigated while monitoring the ^1^H-^15^N HSQC spectra of ^15^N-Adx. Two-dimensional NMR spectra were acquired in the presence of 3-fold and 5-fold excess concentrations of ligand-free and cYY-bound CYP121, along with spectra in the presence of CYP121 containing the R95E_R352E mutation. Similar NMR experiments using ^15^N-Adx have previously been used to demonstrate the presence of multiple contact sites between mitochondrial CYPs and Adx (12, 21, 40). The formation of the protein complex typically leads to differential peak broadening, and interacting surfaces are highlighted by comparing the remaining peak intensities for bound spectra relative to the corresponding intensities for free ^15^N-Adx. The peak ratios resulting from all additions of CYP121 and related line broadening on the Adx are summarized in Figs. S4 and S5, and the ratios resulting from the 5-fold additions of wild-type CYP121 are shown in Figure 8.

Here, the addition of CYP121 resulted in only modest overall line broadening, with between 80% and 90% of the signal remaining relative to the free ^15^N-Adx spectrum, despite additions of significantly higher amounts of the CYP. However, it was confirmed that the modest decrease in intensities reflects the formation of the CYP121-Adx complex since spectra containing the R95E_R352E mutation remained near 100% of the original intensity (see Fig. S3). For experiments that included ligand-free CYP121, peak broadening was also very uniformly distributed (Fig. 8*A*). Residues that are broadened more than one standard deviation from the mean are few, are distributed throughout the Adx structure, and notably do not include residues near ⍺-helix 3, which is the putative Adx recognition site (12, 21, 40). Interestingly, the corresponding experiment in the presence of cYY-bound CYP121 resulted in comparatively more differential peak broadening that was mapped to ⍺-helix one and, notably, near to ⍺-helix 3 as well (Fig. 8*B*). This outcome indicates that the presence of CYP121 substrate results in increased specificity in the interaction between CYP121 and the redox partner. Next, the experiment was repeated using CYP121 in complex with the inhibitor clotrimazole (Fig. 8*C*). In this case, the differential peak broadening near ⍺-helix three that was observed with cYY was no longer present, indicating that modulation of the proximal surface may be a specific response to cYY, rather than a general outcome of ligand-bound CYP121. Taken together, these data highlight the functional relevance of substrate-induced changes to the proximal surface.

## Discussion

The conveyance of structural information through correlated motions or conformation selection plays an important role in cytochrome P450 function. Such allostery contributes to effects across the heme as induced by either substrate or redox protein interactions. The functional consequences of redox protein-induced allostery appear to be events such as repositioning of the substrate within the active site (CYP11A1), alteration of site-specific hydroxylation of the substrate (41, 42), and promotion of either a closed or open CYP structure (P450cam, CYP24A1, CYP11A1, and CYP11B1 fusion) (8, 12, 21, 22, 23, 40, 43); whereas allostery induced from the distal side of the heme regulates the affinity of the CYP-redox protein interaction (23). However, the specific regions of CYP structures that are involved in these long-range adjustments are not well defined.

The current study aimed to investigate functional allostery across the heme in a CYP enzyme. The rationale for the selection of the Mtb enzyme CYP121 was two-fold. First, the robustness of CYP121 production from *E. coli*, combined with the high relative stability of the purified protein, allowed us to record solution NMR spectra at concentrations and buffer conditions that are otherwise not available when investigating mammalian membrane-extracted CYPs. Second, CYP121 has the unique advantage of forming stable distal-to-distal dimers in solution, with a monomer orientation that resembles mammalian enzyme membrane insertion topology. The CYP121 dimer interface is held in place by distal-facing non-polar side chains (Ile-166, Ile-180, Val-373, Val-379) that would otherwise be solvent-exposed. This leads to an arrangement in which the close proximity of the opposite monomer's C-terminal loop region constrains the FG-loop's movement. Importantly, the C-terminal end of the F-helix (Ile-166) and the N-terminal side of the G-helix (Ile-180) form a large part of the interface, while the rest of the F and G α-helices tilt away from the interface. This, again, is very similar to the membrane insertion tilt measured in mammalian enzymes like CYP3A4 (44). Therefore, in our assessment, CYP121 presents an opportunity to investigate allostery in an environment where the motion at the distal side of the molecule is constrained, as is supported by smaller crystallographic B-factors relative to the proximal surface (PDB ID – 3G5H), and therefore is more representative of membrane-bound CYPs than other bacterial enzymes.

**Figure 9:**
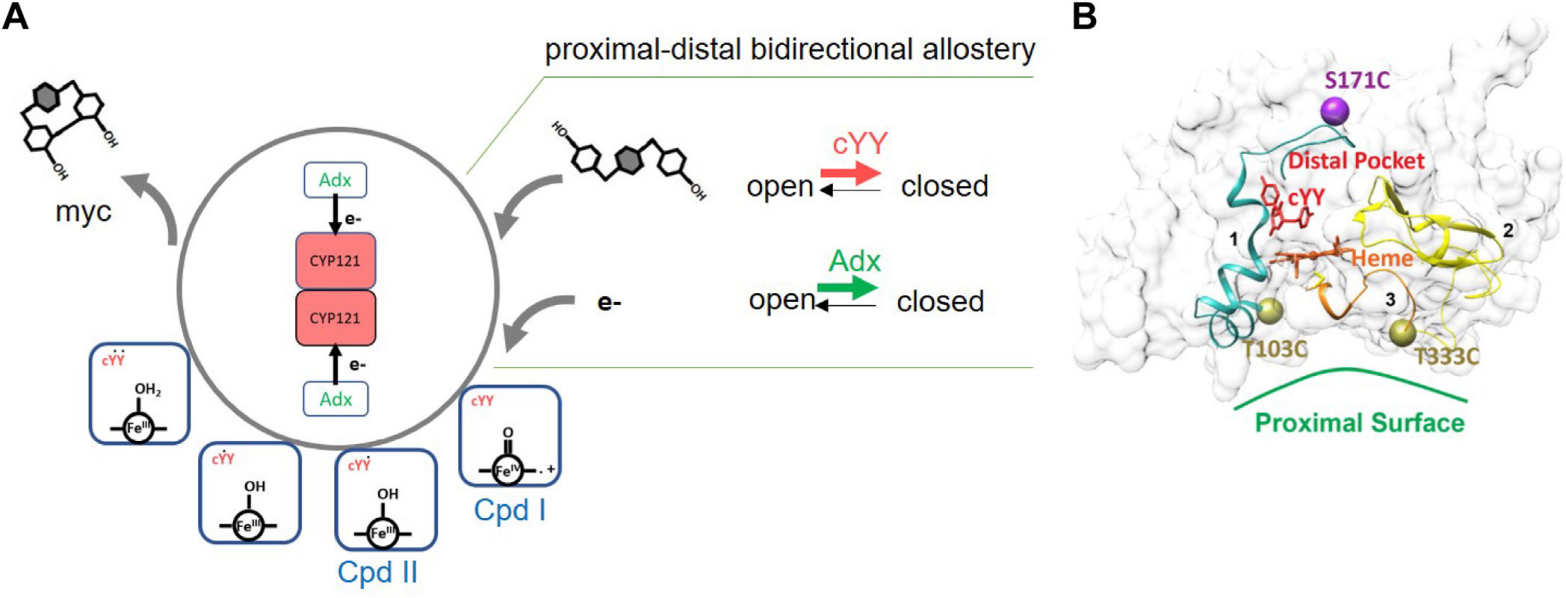
**Candidate allosteric routes for bidirectional communication in CYP121.***A*, overview of CYP121 phenol coupling reaction cycle, with an emphasis on the role of bidirectional allostery induced by substrate and redox partner binding. *B*, route one in *cyan* includes the B-helix, the BC-loop, and connects to the C-helix of the proximal surface and T103C. Route two in *yellow* includes the N-terminal β-strand loop, connected to the meander region and T333C. Route three in *orange* includes the heme and the meander region at T333C. cYY is shown in *red*, and the S171C residue representing the FG-loop is shown in *purple* (*B*).

A remarkable finding from the current study is that upon the addition of Adx, the ^19^F-NMR spectrum of the FG-loop (CYP121_S171C) closely resembles the spectrum of the loop when bound to cYY. In either experiment, the downfield resonance decreases in intensity, while the broad upfield resonance becomes both more intense and narrower in line width. We have previously characterized ligand binding in CYP121 by changes in the ^19^F-NMR spectrum of the FG-loop. With every ligand tested from the previous study, binding resulted in the disappearance of the downfield resonance and a decrease in the slowly exchanging heterogenous conformations of the loop. This downfield resonance is, therefore, now interpreted to represent a form of the open state of the enzyme. Accordingly, we interpret the upfield resonance to represent a form of the closed structure that becomes more structurally homogenous when cYY is bound. In this context, the presence of the redox protein decreases the sampling of the open state and reinforces the FG-loop's closed state. Increasing the proportion of the closed state may also explain what appears to be a decrease in the apparent affinity for cYY (Fig. 7*A*), but is likely due to a change in the rate of substrate binding. Notably, this effect is no longer present when the protein interaction is interrupted (Fig. 7*D*). This finding is consistent with the crystallographic structures of various mitochondrial CYPs. Structures of the cholesterol and corticosteroid metabolizing enzymes CYP11A1 and CYP11B2, both in complex with Adx as fusion proteins, reflect a closed structure (43, 45). Similarly, allosteric modulation of ligand binding induced by Adx in CYP11B1, CYP24A1, and CYP11A1 may also align with promoting a closed CYP conformation. The relationship between the open-to-closed transitions of CYP121 and the overall reaction scheme for the enzyme is highlighted in Figure 9*A*.

This study also reports on long-distance, ligand-induced allostery as detected using site-specific fluorine labeling of two distinct regions on the proximal surface of CYP121. The two labeled sites, ^19^F-T103C and ^19^F-T333C, represent opposite sides of the proximal surface, divided by the heme-coordinating residue Cys-345. Careful selection of these sites was informed first by the similar polarity of threonine to that of cysteine, preserving interactions with the redox protein as much as possible, and second by their likely sensitivity to ligand binding. Structural features that potentially link the distal side of the molecule to either label are summarized in Figure 9*B*. Thr-103 (Route 1) is located at the end of the C-helix and is therefore adjacent to the B′-helix and the BC-loop; a region that is adjacent to the FG-loop borders the active site and is known to be sensitive to ligand binding in other enzymes (8, 9, 23). In CYP121, the BC-loop also contains Asn-85, which forms a hydrogen bond with the diketopiperazine ring of cYY (30). Opposite of this site is Thr-333 of the meander region. While this region lacks well-defined secondary structural elements, five neighboring proline residues suggest some degree of rigidity. The meander region also abuts the A-helix, which is adjacent to the N-terminal β strand loop (Route 2), the latter of which forms part of the active site “roof” and is therefore also theorized to be affected by an open-to-close transition. Thr-333 also serves as a marker of structural changes that include the heme itself (Route 3), due to its connection to the axial Cys-345. Here, it was observed that the NMR spectrum of ^19^F-T333C of the meander region does undergo ligand-induced perturbations (Figs. 4 and 5). However, these occurred entirely in step with ligand-induced changes in the heme spin state. While this does not completely preclude structural changes in this region, it does suggest that many of the effects observed are derived from paramagnetically induced pseudocontact shifts. In contrast, ^19^F-T103C of the C-helix undergoes NMR spectral perturbations that are not solely explained by the heme spin state. The addition of cYY, in particular, resulted in a pronounced new peak that did not resemble salt-induced manipulation of the spin state (Fig. 4*A*). An additional, albeit unintended, indicator that this site is in a functionally important region is that catalytic activity for T103C is reduced relative to the native enzyme and to T333C (Fig. 3). Therefore, we conclude that substrate and ligand-induced allostery in CYP121 involves the C-helix, but not the meander region, of the proximal surface. The presence of similar perturbations in ^19^F-N84C of the B-helix, also contained within Route 1, further supports this path (Fig. 6). This finding has parallels to the study of allostery in P450cam from *P. putida*, in which the presence of valine and proline sequential residues on the B′-helix are responsible for a slow *trans*-*cis* isomerization that connects opposite ends of the structure (46, 47). Interestingly, CYP121 also contains a Val-78, Pro-79 sequence just prior to Asn-84. Therefore, a similar isomerization in CYP121 warrants further investigation.

With respect to mammalian CYPs, to our knowledge, there is relatively less known regarding how the C-D region is affected upon ligand binding. A previous study focused on CYP24A1 relied on differential chemical cross-linking coupled to mass spectrometry to identify a region at the C-D α-helices that was only affected when Adx and substrate were present together (21). In a more recent study, the Atkins group utilized hydrogen-deuterium exchange combined with mass spectrometry to highlight a ligand-induced effect on several regions of CYP3A4, notably also including the C-D helices (48). In general, given the results of the current study combined with the proximity of the C-D helical surface to the B-helix, we surmise that this route represents a conserved allosteric network in CYP structures.

Remarkably, the conserved nature of such allostery is also indicated by the Adx-detected NMR studies (Fig. 8). Here, only the addition of cYY resulted in the recognizable peak broadening pattern expected from the interaction. However, the fact that these proteins are derived from organisms that belong to distinct kingdoms of life, yet still display increasingly specific molecular recognition when in the presence of the CYP substrate, indicates the communication of a fundamental change in CYP structure (such as open to closed transitions) that is not species or isoform-specific.

Finally, while investigating the functional role of long-range allostery, we found that the CYP121 complex with redox protein actually decreases the apparent affinity for cYY (34), an effect that was removed when the protein interaction was disrupted (Fig. 7*A*). This was predicted based on the finding that redox protein induced a closing of the active site FG-loop. Promoting a closed structure at this stage is logical within the context of CYP function, as it would likely prevent substrate dissociation prior to catalysis. In such a model, dissolution of the CYP-redox protein complex following the second electron transfer may be required to re-open the active site and dissociate the reaction product. We would also like to note that while we surmise that Adx likely induces an open-to-close transition in mammalian CYPs, the allosteric effect on ligand binding may be CYP and ligand-specific. For example, Adx is now known to potentiate ligand binding in CYP11B1, CYP11B2, and CYP24A1, which is opposite from our findings for CYP121. Therefore, the particular effect on the interaction with cYY may be unique to CYP121.

Overall, this work demonstrates that the redox protein interaction promotes a closed conformation in a bacterial CYP that serves as a model system for motionally constrained (mammalian) enzymes. The finding that allostery is bidirectional is not surprising. However, this work outlines a path for this allostery that connects the FG-loop of CYP121 with the C-helix of the proximal surface in what may represent a conserved network in the structurally homologous CYP protein fold, while also informing function in an essential Mtb CYP.

## Experimental procedures

### Protein expression and purification

Wild-type and mutant CYP121 protein was generated in *E. coli* as described previously (34). Briefly, bacterial cultures were grown in Luria Broth at 200 rpm shaking and 37 °C until reaching an optical density reading of 0.8. Expression of protein was induced with 1 mM of isopropyl ß-D-1-thiogalactopyranoside and 0.5 mM δ-aminolevulinic acid. Cultures in the growth phase were incubated for 48 h at 22 °C with shaking at 160 rpm. Harvested cell pellets were stored in lysis buffer (50 mM Tris-HCl, 300 mM NaCl, pH 7.4) at −80 °C until use. Chemical cell lysis was performed using sonication at 30 s intervals for a total of 3 min. Soluble protein was separated from cell debris by centrifugation at 100,000*g* for 45 min. The supernatant was passed over a pre-equilibrated charged nickel nitroacetic acid (Ni-NTA) resin with loading buffer (50 mM Tris-HCl, 300 mM NaCl, 5 mM imidazole, pH 7.4), washed with 5X column volumes of wash buffer (50 mM TrisHCl, 300 mM NaCl, 50 mM imidazole, pH 7.4), and eluted with 50 mM TrisHCl, 300 mM NaCl, 100 mM imidazole, pH 7.4. Elution fractions were pooled based on an absorbance ratio (A_420_/A_280_) of 1.2 or above, concentrated, and passed through a pre-equilibrated 180 ml size exclusion column with 50 mM Tris-Cl, 300 mM NaCl, pH 7.4. Point mutations were generated at Genscript and purified similarly to the wild-type protein. Adrenodoxin Reductase (AdR) and Adx were generated as described previously (12, 21).

### ^19^F labeling of CYP121

Pooled eluent from Ni-NTA affinity chromatography was diluted to 2 μM protein in 50 mM Tris HCl, pH 7.4, and 300 mM NaCl supplemented with 10 mM 3-Bromo-1,1,1-trifluoroacetone (BTFA) and 5 mM DTT and incubated overnight at 4 °C. Unreacted BTFA and DTT was removed by size exclusion chromatography. The protein was then exchanged into the NMR buffer (50 mM potassium phosphate, pH 7.4, 50 mM NaCl, and 10% D_2_O) using a 10 kDa molecular weight cut-off filter (Millipore).

### 1D NMR data acquisition and analysis

Labeled protein was aliquoted into separate NMR samples at 250 μM and 450 μl volumes. Three-fold or five-fold ligands and five-fold bovine Adx were added before transfer into NMR sample tubes. Binding of ligands was confirmed by observing the Soret peak shift using UV visible spectroscopy (Fig. S1). NMR spectra were acquired on an Agilent 400 MHz spectrometer at 25 °C, with a 30° pulse angle, 1-s delay, and −84 ppm transmitter offset frequency. Each experiment consisted of 10,000 scans. The data were processed and analyzed in TopSpin, version 4.1.1.

### 2D NMR data acquisition and analysis

Uniformly labeled ^15^N-Adx was expressed in minimal media containing ^15^N ammonium chloride, as described previously (12), and purified by standard Ni-NTA and size exclusion chromatography. In order to minimize masking of charges for the electrostatic interactions between the proteins, samples were prepared in the same low salt NMR buffer as described for ^19^F experiments, but using ^15^N-Adx at 100 μM and combined with excess CYP121 in ratios of 1:3 and 1:5. For substrate-bound samples, cYY was added to CYP121 in the stoichiometric ratio of 1:1. After 10 min on ice, cYY-binding was confirmed using UV visible spectroscopy. For the inhibitor-bound sample, excess clotrimazole was added to CYP121 to saturate it and binding was confirmed with a Soret shift from 415 nm to 421 nm (Fig. S2). Subsequently, 10% D_2_O was added to each sample and 2D ^1^H-^15^N HSQC NMR data were acquired on a Bruker Avance III 800 MHz instrument and using 32 scans and 128 fids at 25 °C. Raw data were processed using NMRPipe (49) and analyzed using VNMRj (50). The peak intensities corresponding to each assigned amide were quantified from the processed spectra. The values were expressed as a ratio of remaining peak intensity using the following formula: Intensity (remaining) = Intensity (CYP added)/Intensity (free ^15^N-Adx) (Fig. S4). Differential peak broadening was then calculated for residues in which intensity was affected more than one standard deviation from the mean of all residues.

Data were analyzed using VNMRj. The peak intensities corresponding to each assigned amide were quantified from the processed spectra. The values were expressed as a ratio of remaining intensity using the aforementioned formula. These values identified differential peak broadening using one full standard deviation from the mean of the remaining intensities. The assigned amides quantified with lower intensity than mean-1SD were highlighted with a red circle, and corresponding residues were also highlighted in red on the protein structure (Fig. S5). For all structural images, we used UCSF Chimera (51).

### Ligand binding assays

Ligand binding was measured using UV-visible spectroscopy on a dual-beam Shimadzu 2700 spectrophotometer. Stocks of cYY (a gift from the laboratory of Dr Claire Simons, Cardiff University) and azole inhibitors (GoldBio) were prepared in DMSO to 200 mM final stock concentration. Compounds were titrated into a 1 μM solution of CYP121 in 50 mM Tris-Cl and 50 mM NaCl, pH 7.4, in a 1 cm quartz cuvette. All titrations were carried out in triplicate at 25 °C with an 8-min incubation period between each titration point to reach binding equilibrium. Dissociation constants were calculated as done previously by plotting ligand concentrations against the absolute value of the maxima and minima of the absorbance difference spectra. Data fitting was carried out in Prism GraphPad v7.05 and using a one-site binding mode equation as was reported previously (34). For samples containing both CYP121 and Adx, the Adx signal was subtracted prior to titration of cYY.

### CYP121 activity assays

CYP121 functional assays were prepared using Adx and adrenodoxin reductase (AdR) from bovine. The protocol is as recently described (34). The reaction mixture contained 5 μM CYP121, 15 μM Adx, 5 μM AdR, and 150 μM substrate in 150 μl total volume of 50 mM Tris HCl (pH 7.4). The reaction mixture was preincubated for 5 min at 30 °C, and the reaction was initiated upon the addition of 1 mM NADPH. The reactions were quenched after 0, 15, 30, and 60 min by the addition of 22.2 μl of 20% nitric acid in 200 μl reaction mixture and 5 μM L-tryptophan was added as a standard. 50 μl of the quenched reaction was combined with 50 μl of acetonitrile and centrifuged at 10,000 rpm for 30 min. Following centrifugation, 20 μl of the supernatant was diluted into 180 μl of 10% acetonitrile and 0.1% formic acid in water. Reactions were carried out in triplicate and samples were resolved on a Poroshell 120 EC-C18 column (4.6 mm × 250 mm) (Agilent) on an Agilent 1260 Infinity II liquid chromatography system. The formation of the product peak was quantified as a ratio of the internal standard L-tryptophan peak. One-way ANOVA, two-way ANOVA, or *t* test performed all the statistical analysis. All the significant values were considered only if *p* < 0.05.

### Chemical cross-linking

Purified wild-type CYP121 and mutant proteins were incubated with Adx under low-salt conditions (10 mM potassium phosphate buffer, pH 7.4) and in the presence of the zero-length cross-linker EDC (2 mM working concentration) (Thermo Scientific). CYP121 (5 μM) was combined with 100 μM Adx or its mutants in a 20 μl reaction volume. After a 2-h incubation at 25 °C, the reaction was terminated by the addition of an equal volume of Laemmli loading dye (Bio-Rad). The samples were then resolved by gel electrophoresis and stained with Coomassie Blue.

## Data availability

All processed NMR spectra are available upon request.

## Supporting information

This article contains supporting information.

## Conflict of interest

The authors declare that there are no conflicts of interest with the contents of this article.
