# Supplementary material for: Structural basis of bidirectional allostery across the heme in a cytochrome P450 enzyme
Source: J Biol Chem. 2023 Jun 29;299(8):104977. doi: 10.1016/j.jbc.2023.104977 (PMC10416055; doi:10.1016/j.jbc.2023.104977)
Supplement: Supporting Figures S1–S5 [file mmc1.docx]

**SUPP0RTING INFORMATION**

**Structural basis of bidirectional allostery across the heme in a cytochrome P450 enzyme**

Amit Kumar^1^ & D. Fernando Estrada*^1^

^1^Department of Biochemistry, Jacobs School of Medicine and Biomedical Science, University at Buffalo, Buffalo, NY 14203, USA.

*Corresponding author, Email ID - dfestrad@buffalo.edu

**Table of Contents**

**SUPPLEMENTAL FIGURES** ………………………………………………………….……...S-1-S-6

Figure S1 **Spectrum of ligand free (blue) and azole-bound CYP121 by UV visible spectroscopy.** ………………………………………………………….……………….……. S-2

Figure S2 **Spectrum of ligand free (blue), cYY bound (red) and clotrimazole-bound CYP121 (salmon red) by UV visible spectroscopy.** …………………….……………………..…. S-3

Figure S3 **Functional characterization and the azole-induced effect on ^19^F-N84C of CYP121 ……………..**………………………………………………………………………………..…….S-4

Figure S4 **Amide peak intensity plot ratios derived from 15N-Adx HSQC spectra with and without CYP121.**………………………… …………………………………………………… S-5

Figure S5 **The line-broadened residues obtained from 2D HSQC NMR data analysis are highlighted in red on bovine Adx (PDB 1AYF).**…………………………………………. S-6

SUPPLEMENTAL FIGURES


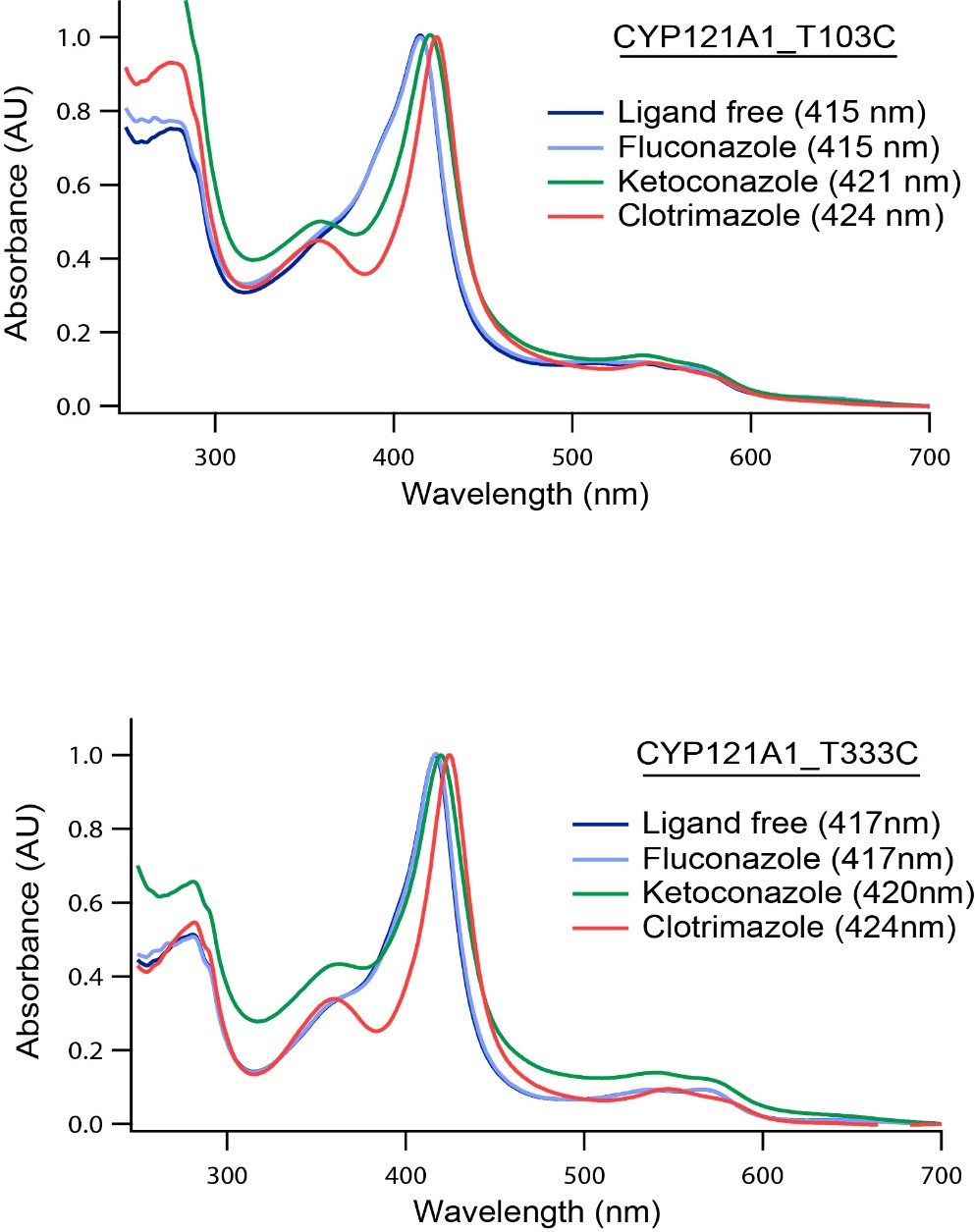


**Figure S1.** **Spectrum of ligand free (blue) and azole-bound CYP121 by UV visible spectroscopy.** NMR samples were measured directly following preparation and prior to NMR acquisition in order to confirm Soret peak value upon ligand-binding.


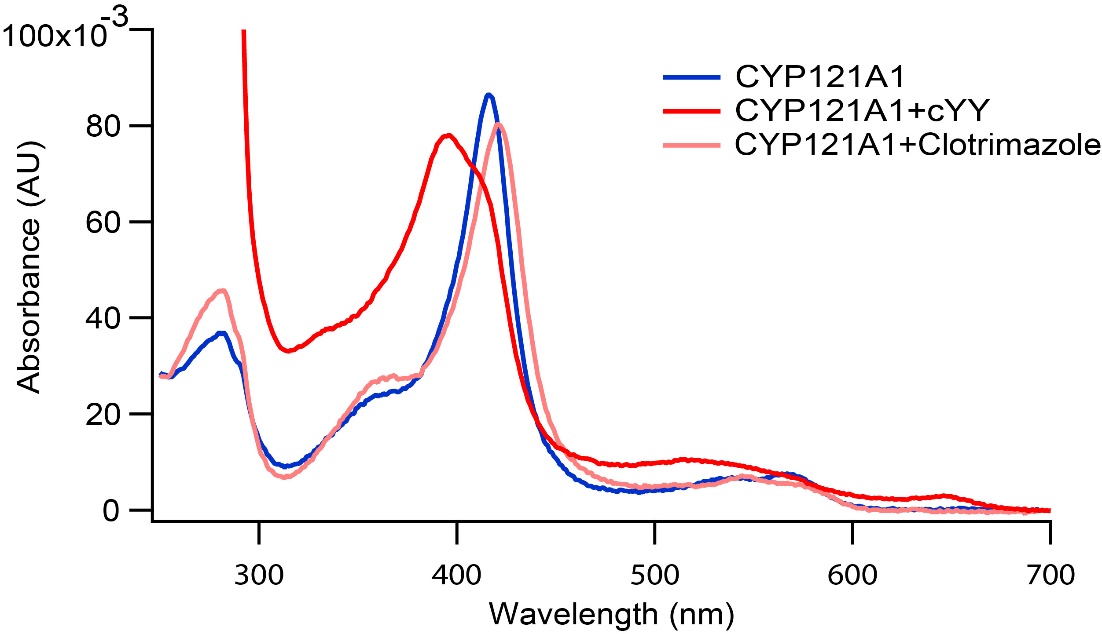


**Figure S2.** **Spectrum of ligand free (blue), cYY bound (red) and clotrimazole-bound CYP121 (salmon red) by UV visible spectroscopy.** CYP121 was measured just prior to addition into NMR samples of 15N-Adx for 1H-15N HSQC acquisition.


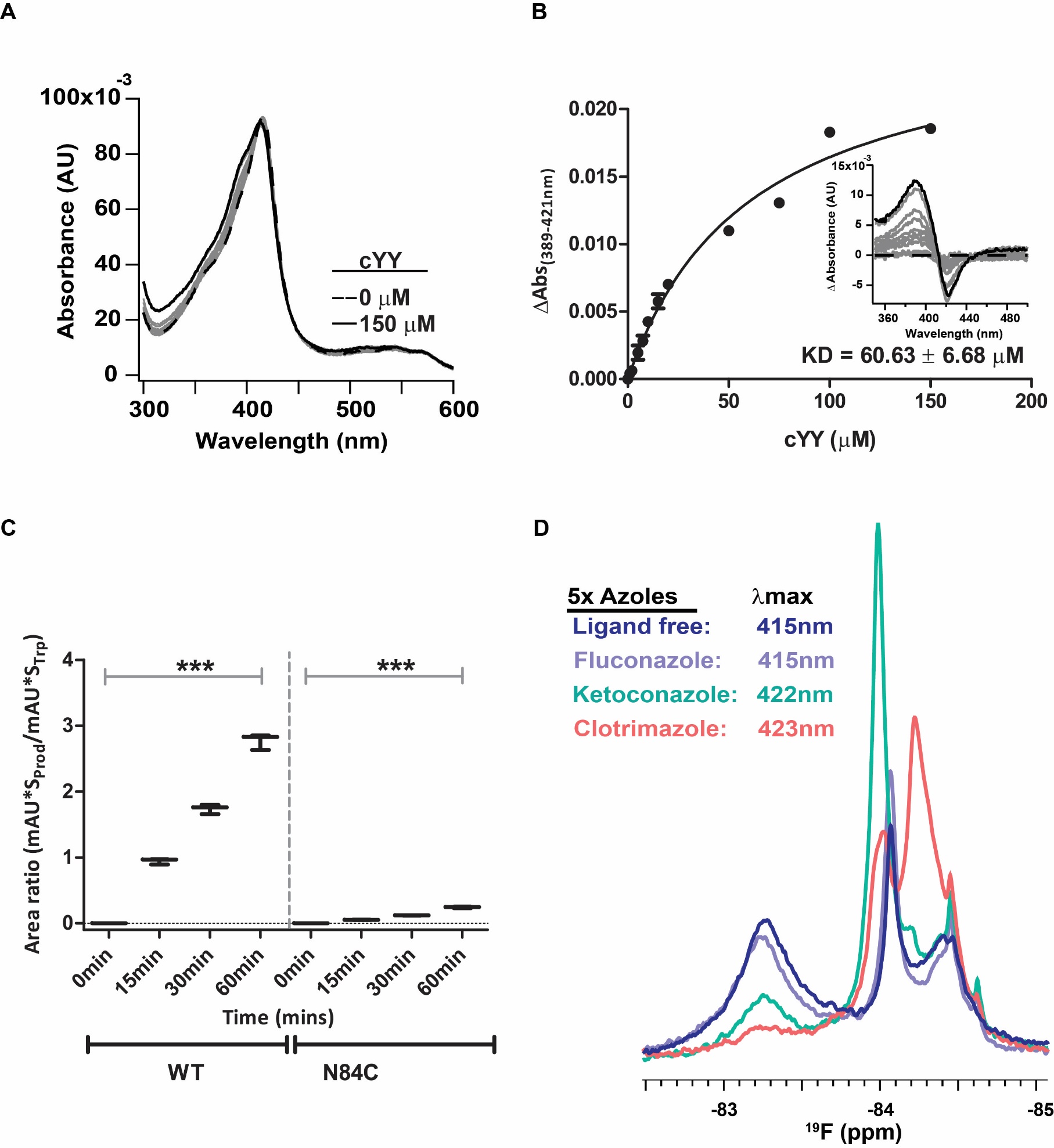


**Figure S3.** **Functional characterization and the azole-induced effect on ^19^F-N84C of CYP121.** The N84C mutation results in loss of cYY binding (A and B) as compared to wild-type protein. Compromised binding also results in a significant loss of activity (C). However, changes in the 19F-NMR spectra at this site do not occur in step with increasing values of the Soret peak (D), thus indicating that ligand-induced changes in structure at this site nonetheless occur.


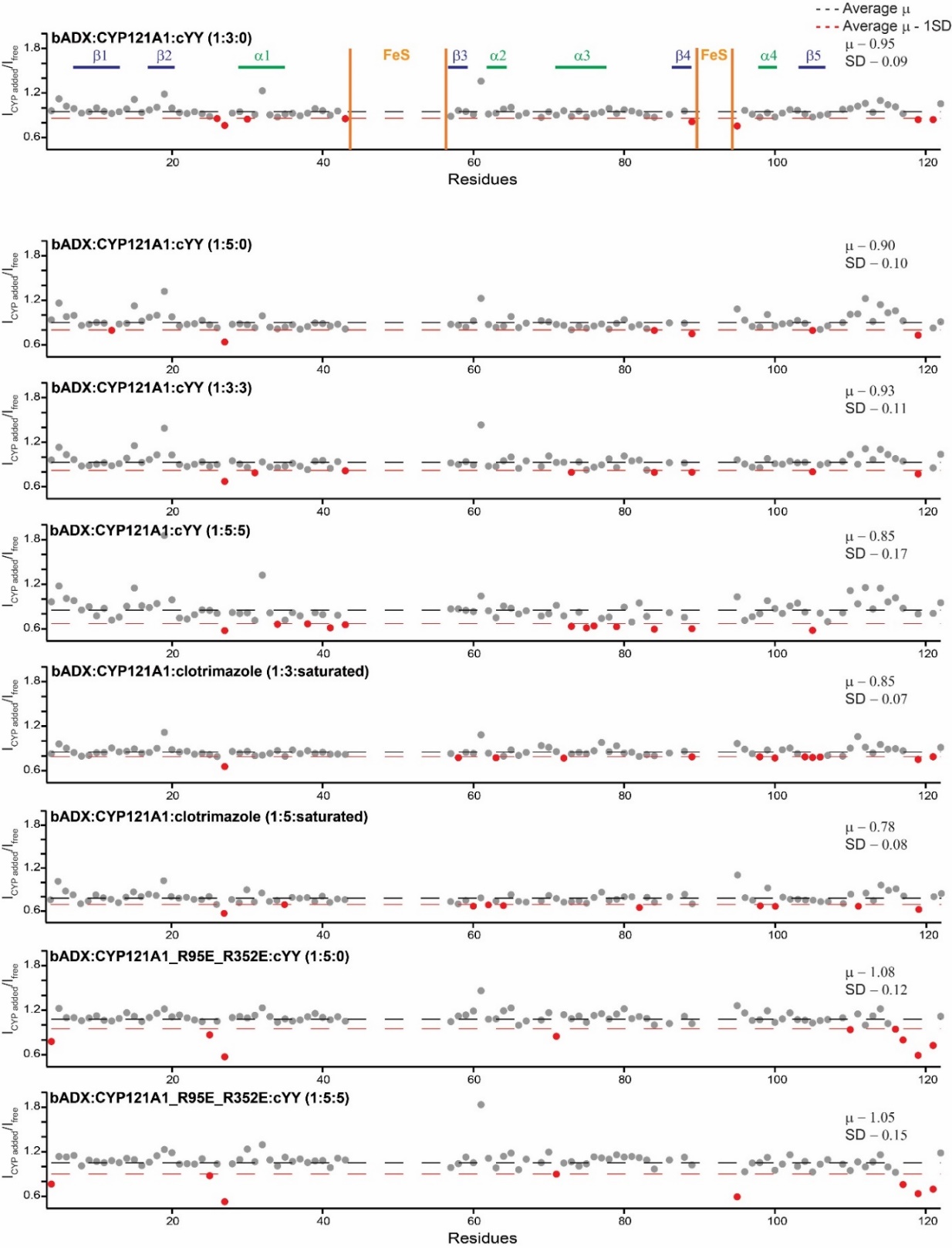


**Figure S4.** **Amide peak intensity plot ratios derived from ^15^N-Adx HSQC spectra with and without CYP121.** Average loss of intensity resulted in approximately 80% of original signal, with the exception of the R95E_R352E, in which near 100% of the original 15N-Adx signal remains.


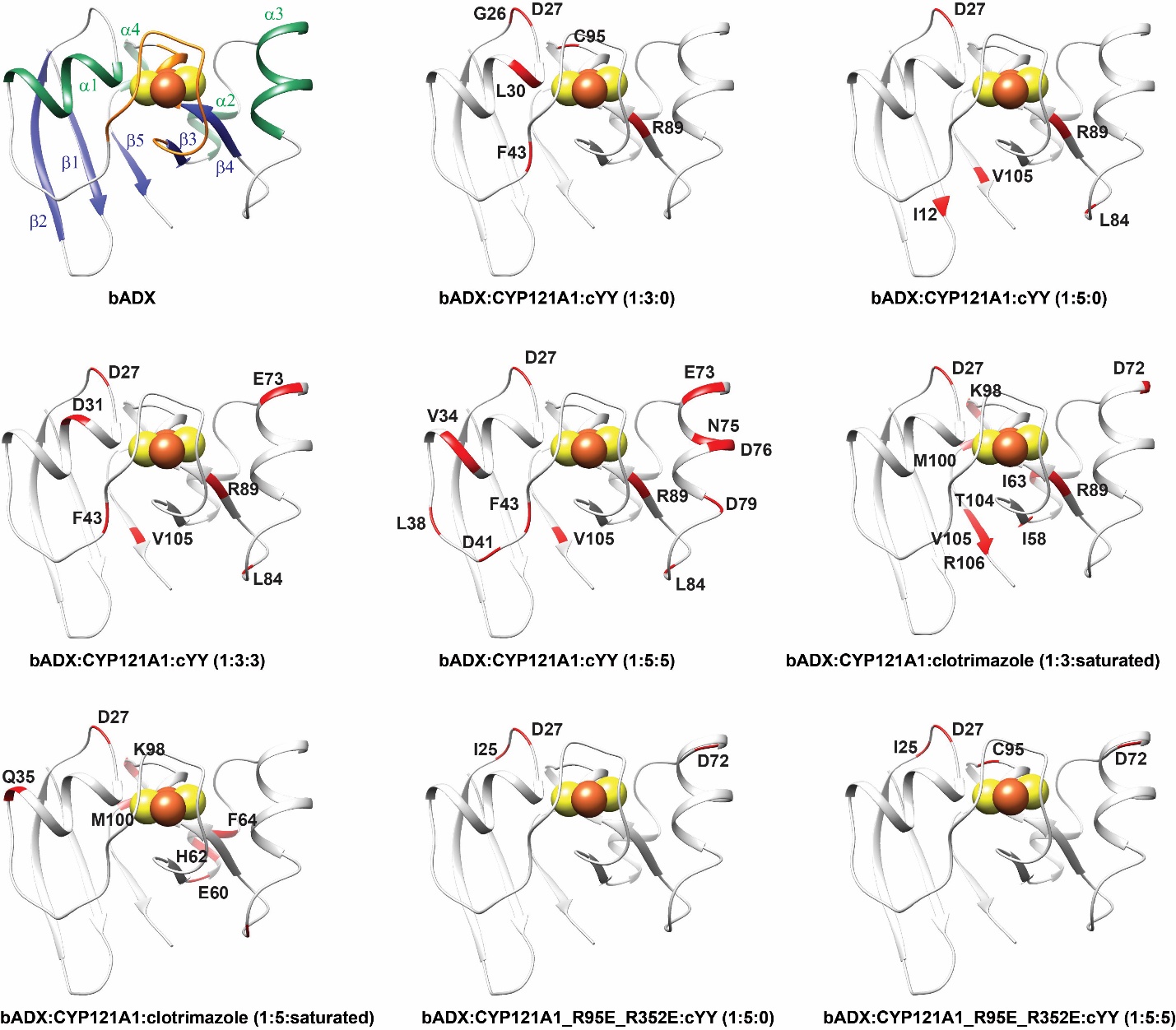


**Figure S5. The line-broadened residues obtained from 2D HSQC NMR data analysis are highlighted in red on bovine Adx (PDB 1AYF).** Experiment titles correspond to the intensity plots in Figures S3.
